# Postoperative Complications, In-Hospital Mortality and 5-Year Survival After Surgical Resection for Patients with a Pancreatic Neuroendocrine Tumor: A Systematic Review

**DOI:** 10.1007/s00268-015-3328-6

**Published:** 2015-12-10

**Authors:** Anneke P. J. Jilesen, Casper H. J. van Eijck, K. H. in’t Hof, S. van Dieren, Dirk. J. Gouma, Els J. M. Nieveen van Dijkum

**Affiliations:** Department of Surgery, Academic Medical Center, Meibergdreef 9, P. O. Box 22660, 1105 AZ Amsterdam, The Netherlands; Department of Surgery, Erasmus Medical Center, Rotterdam, The Netherlands; Department of Methodology and Statistics Clinical Research Unit, Academic Medical Center, Amsterdam, The Netherlands

## Abstract

Studies on postoperative complications and survival in patients with pancreatic neuroendocrine tumors (pNET) are sparse and randomized controlled trials are not available. We reviewed all studies on postoperative complications and survival after resection of pNET. A systematic search was performed in the Cochrane Central Register of Controlled Trials, MEDLINE and EMBASE from 2000–2013. Inclusion criteria were studies of resected pNET, which described postoperative complications separately for each surgical procedure and/or 5-year survival after resection. Prospective and retrospective studies were pooled separately and overall pooled if heterogeneity was below 75 %. The random-effect model was used. Overall, 2643 studies were identified and after full-text analysis 62 studies were included. Pancreatic fistula (PF) rate of the prospective studies after tumor enucleation was 45 %; PF-rates after distal pancreatectomy, pancreatoduodenectomy, or central pancreatectomy were, respectively, 14–14–58 %. Delayed gastric emptying rates were, respectively, 5–5–18–16 %. Postoperative hemorrhage rates were, respectively, 6–1–7–4 %. In-hospital mortality rates were, respectively, 3–4–6–4 %. The 5-year overall survival (OS) and disease-specific survival (DSS) of resected pNET without synchronous resected liver metastases were, respectively, 85–93 %. Heterogeneity between included studies on 5-year OS in patients with synchronous resected liver metastases was too high to pool all studies. The 5-year DSS in patients with liver metastases was 80 %. Morbidity after pancreatic resection for pNET was mainly caused by PF. Liver resection in patients with liver metastases seems to have a positive effect on DSS. To reduce heterogeneity, ISGPS criteria and uniform patient groups should be used in the analysis of postoperative outcome and survival.

## Introduction

Given the rarity of pancreatic neuroendocrine tumors (pNET), well-designed randomized controlled trials on surgical treatment for pNET are not available [1–3]. Most studies are cohort studies or case reports and therefore the level of evidence in studies on surgical treatment of pNET is limited to level III.

Studies on postoperative complications and in-hospital mortality often describe pNET as part of a larger study population. These studies include patients with pancreatic ductal adenocarcinoma, intraductal papillary mucinous neoplasm (IPMN), chronic pancreatitis, pancreatic adenomas as well as pNET [4–6]. These diagnoses may influence the postoperative complication rate and operative mortality. It is well known that patients with pancreatitis have a lower postoperative pancreatic fistula rate compared to non-pancreatitis patients [7]. Furthermore, postoperative complications after pancreatic surgery for pNET are influenced by the type of surgery, such as pancreatoduodenectomy, distal pancreatectomy, central pancreatectomy, or enucleation [8–11]. Studies analyzing postoperative complications caused by the different surgical procedures in patients with pNET are limited.

Survival of pNET patients is mainly affected by metastasis found at the time of diagnosis. The overall 5-year survival of non-functional pNET in patients with distant metastases (M1) is 43 % with a median survival of 23 months In contrast, patients with resected functional pNET without metastases (M0) have a survival rate of 90–100 % [2, 3]. Survival is often presented by tumor stages but different staging systems are used, e.g., American Joint Committee on Cancer (AJCC) staging or European Neuro Endocrine Tumor Society (ENETS) staging system [12, 13]. Another difficulty in analyzing survival of patients with pNET after resection is the inclusion of non-hereditary and hereditary patients in the same cohort. Survival outcome of patients Multiple Endocrine Neoplasia type 1 (MEN-1) or Von Hippel–Lindau (VHL) disease may be influenced since these tumors are often early diagnosed and indication for the surgical treatment can be different [3].

Considering the limitations of most studies as summarized, the aim of this study was to systematic review all studies on postoperative complications and 5-year survival in patients with resected pNET.

## Methods

### Search methods and identification of studies

All types of study, including cohort, case-control or case series and languages, were included. Inclusion period ranged from January 2000 till December 2013. Studies before 2000 were not included. In 2000, the WHO classification was introduced and clearly defined the phenotypes of NETs and their clinicopathological conditions. In order to reduce ambiguities and heterogeneity on pathological origin from the included studies, the time for inclusion was from 2000 to 2013 [14]. The Cochrane Central Register of Controlled trials (CENTRAL) in the Cochrane Library, MEDLINE and EMBASE were searched for studies. Also the references of the identified studies were searched to identify suitable studies.

The search strategy was supervised by the local librarian and the query terms “neuroendocrine tumor”, “carcinoid”, “pancreas”, “foregut”, “pNET”, “GEP-NET”, “pancreatoduodenectomy”, “enucleation”, “pancreatectomy”, “complications”, “fistula”, “bleeding”, “delayed gastric emptying”, “survival” or every possible variants of these terms were used. Two authors (APJJ, EJMND) independently reviewed all included studies on title and abstract and later on full text.

Inclusion criteria were all studies on resected pNET in which the postoperative complications, in-hospital mortality or survival after surgical resection was described. Postoperative complications were defined as pancreatic fistula, delayed gastric emptying, bleeding, and mortality as in-hospital mortality after resection. Finally, at least 10 patients with a pNET had to be included in the study to reduce bias and heterogeneity and to enhance scientific relevance. Studies were scored as invalid if the patients were analyzed as a part of a larger cohort of none-pNET and the data of the patients with a pNET could not be extracted from full-text analysis. Also, if not all described patients had undergone surgery and/or the resected patients have not been described separately or if studies described the postoperative complications or in-hospital mortality of the entire group and not specific after one surgical procedure, studies were scored as invalid. Finally, in order to improve homogeneity, studies were excluded from the 5-year survival analysis if all the patients of the study were affected with the MEN-1-syndrome/VHL disease or if all the included patients in the study had liver metastasis at time of surgery.

### Data collection and statistical analysis

After screening on title and abstract, a full-text screening was performed to determine if the studies fulfilled the inclusion criteria. Data of postoperative complications, in-hospital mortality and survival were extracted. If possible, the complications were scored according the ISGPF/S criteria [15–17]. An overall (grade A/B/C) pancreatic fistula rate and if possible a grade B/C pancreatic fistula rate was calculated. If the grade B/C pancreatic fistula rate was not described in detail, then that study was only included in the overall pancreatic fistula proportion analysis. The same yields for delayed gastric emptying and postoperative hemorrhage. The variables of the postoperative complications and in-hospital mortality were analyzed for each surgical procedure. Studies on survival were only included if the overall 5-year survival and/or the 5-year disease-specific survival after curative resection could be extracted in patients with and/or without curative resected liver metastases. No strict definitions of a curative resection were enforced. If the survival was analyzed based on resection margins, the R0 resection margin was used.

Postoperative complications, in-hospital mortality and 5-year survival were given in proportions with a 95 % confidence interval (CI) and a meta-analysis of these proportions was performed with R [18]. The random effects model was used for expected heterogeneity. The *I*^2^ statistics was used to measure the consistency between the studies in the meta-analysis. If the *I*^2^ statistics was above 75 %, the heterogeneity was considerable and the results of proportion analysis were not suitable for a meta-analysis [19–21]. In order to make a distinction in the quality of the studies, prospective and retrospective studies were analyzed separately. From all the prospective and retrospective studies an estimated pooled proportion was calculated and if the *I*^2^ statistics were both below 75 %, all studies were pooled in an overall proportion.

### Assessment of risk of bias

For the assessment of the risk of bias, the methodological index for non-randomized studies (MINORS) was used [22]. The MINORS contains 8 items: clear stated aim, inclusion consecutive patients, prospective data collection, endpoints appropriate to aim, unbiased assessment of the endpoint, appropriate follow-up period, loss to follow-up <5 % and prospective calculation of study size. Based on these eight items, the included studies will be scored to a 3-point scale from 0 to 2. An item scored 0 if the item was not reported. An item scored 1, if it was reported but inadequate and an item scored 2 if it was reported and adequate. The ideal total score would be 16. An appropriate follow-up for the studies included in the survival analysis was at least 40 months. If it was not exactly described whether all the patients were included in the follow-up, the study scored 1 point in “lost to follow-up”.

## Results

**Fig. 1:**
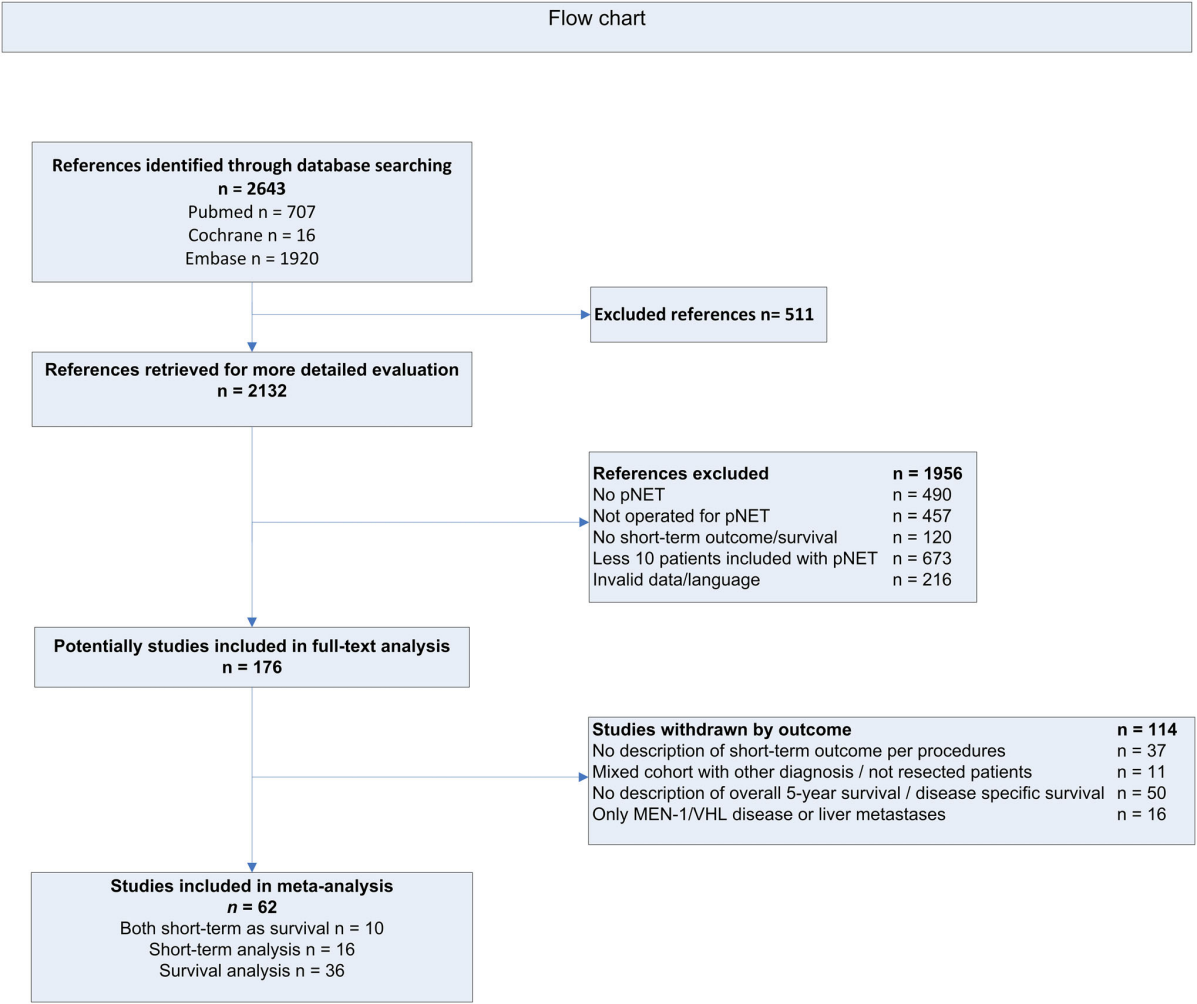
Flow Chart of the search strategy

A total of 2643 studies were identified through searching the different databases, including Cochrane Central Register of Controlled trials (CENTRAL) in the Cochrane Library, MEDLINE and EMBASE. A total of 511 duplicate studies were excluded, as depicted in Fig. 1, therefore 2132 references were suitable for further assessment. Of all these references, 1956 were excluded because they did not meet the inclusion criteria or the studies were invalid. Initially 176 studies were included in the full-text search and after these articles looked through, 114 studies were withdrawn by their outcome. Finally, 62 studies were included in this meta-analysis, 10 studies for postoperative complications, in-hospital mortality and survival analysis, 16 studies for only postoperative outcome analysis and 36 for only survival analysis, as depicted in Fig. 1.

### Postoperative complications

#### Pancreatic fistula

**Fig. 2:**
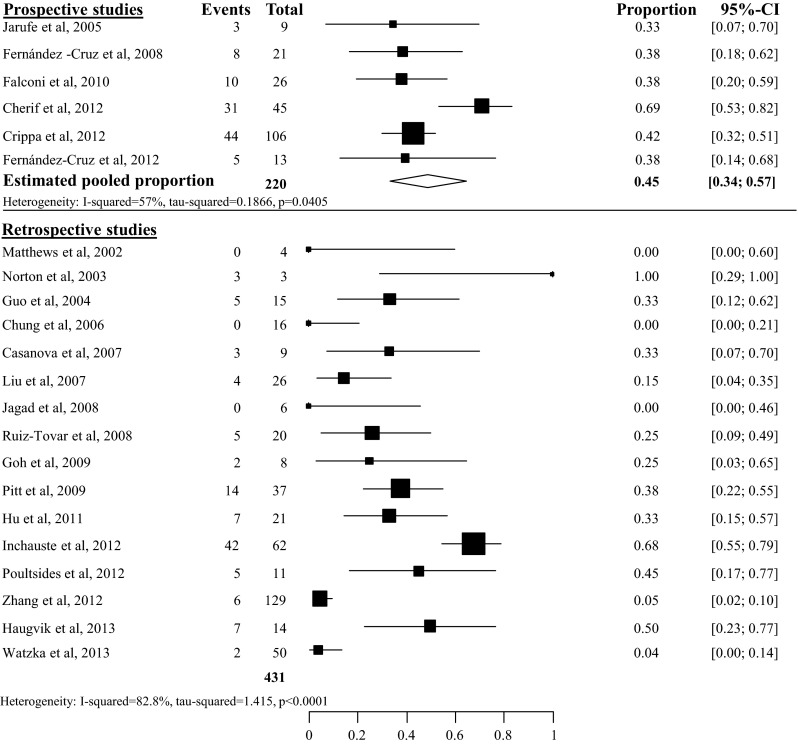
Overall pancreatic fistula rate after tumor enucleation

**Fig. 3:**
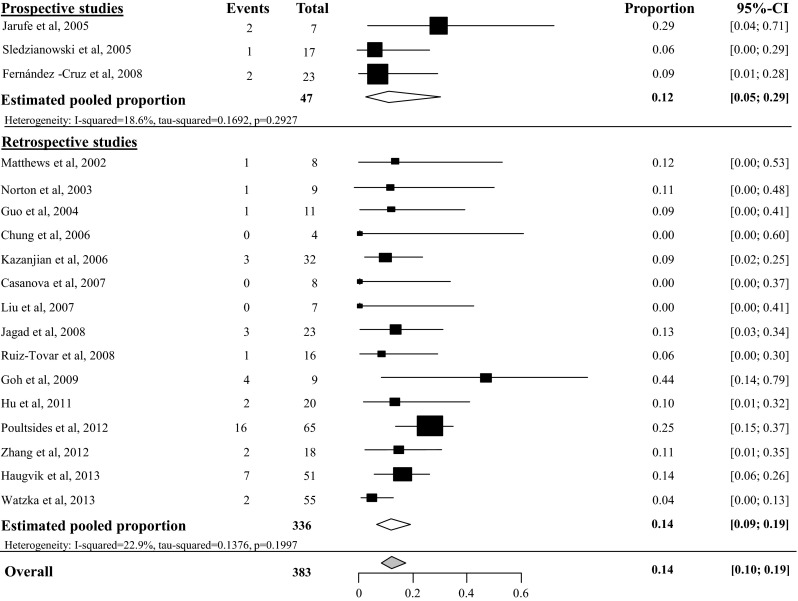
Overall pancreatic fistula rate after distal pancreatectomy

**Fig. 4:**
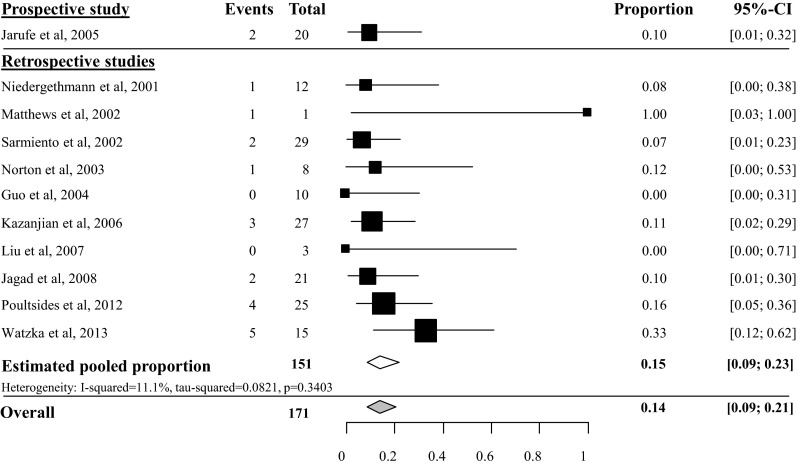
Overall pancreatic fistula rate after pancreatoduodenectomy

Estimated pooled pancreatic fistula (PF) rate after tumor enucleation was 45 % (95 % CI 34–57 %, *I*^2^ 57 %), based on 6 prospective studies with 220 included patients [23–28]. Heterogeneity of the 16 retrospective studies was too high to pool all 22 studies, as depicted in Fig. 2 [29–44]. Overall PF rate grade B/C after tumor enucleation was 27 % (95 % CI 19–37 %), based on 8 studies with a total of 324 included patients [24, 25, 27, 28, 38, 40, 43] (see appendix Fig. 13). Overall PF rate after distal pancreatectomy was 14 % (95 % CI 10–19 %), based on 18 studies with a total of 383 included patients, as depicted in Fig. 3 [23, 24, 29–37, 39, 41–44, 45, 46]. The overall grade B/C PF rate after distal pancreatectomy was 8 % (95 % CI 2–35 %), based on 2 studies with a total of 74 included patients [24, 43] (see appendix Fig. 14). Overall PF rate after pancreatoduodenectomy was 14 % (95 % CI 9–21), based on 11 studies with a total amount of 171 included patients as depicted in Fig. 4 [23, 29–31, 34, 35, 41, 44, 46–48]. None of these studies described grade B/C PF rate in detail. Overall PF rate after central pancreatectomy was 58 % (95 % CI 41–73 %), based on four studies with a total of 56 included patients (see appendix Fig. 15) [25, 28, 34, 41]. Two studies described grade B/C PF rate ranging from 12 to 41 % (see appendix Fig. 16). Heterogeneity was too high to perform a pooled meta-analysis (*I*^2^ 77 %) [25, 28].

#### Delayed gastric emptying

**Fig. 5:**
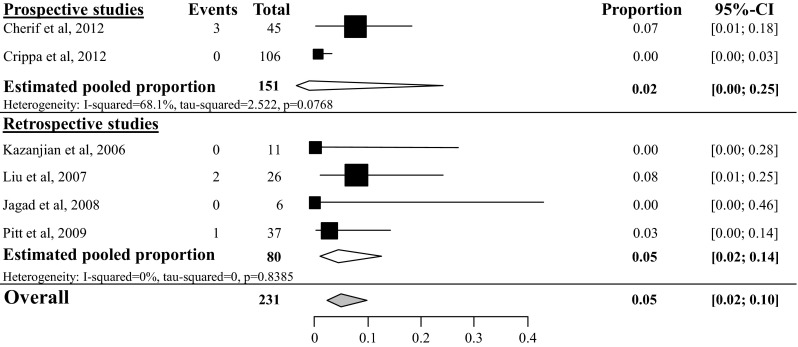
Overall delayed gastric emptying rate after tumor enucleation

**Fig. 6:**
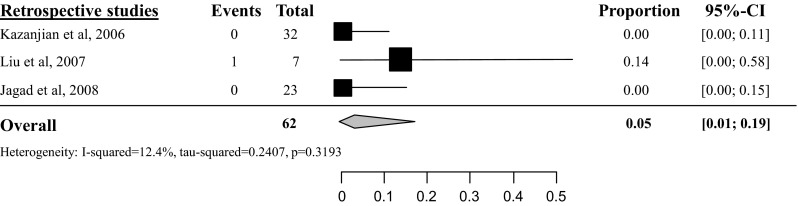
Overall delayed gastric emptying rate after distal pancreatectomy

**Fig. 7:**
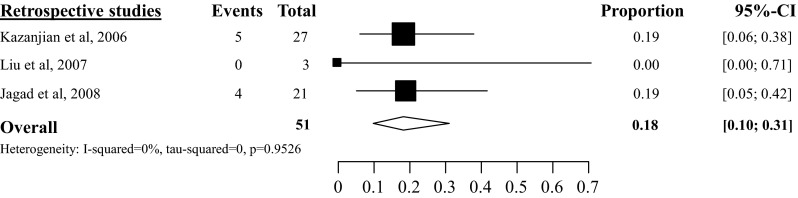
Overall delayed gastric emptying rate after pancreatoduodenectomy

Delayed gastric emptying (DGE) was rarely reported and only the overall DGE rate was analyzed since none of the included studies made a distinction based on the ISGPS criteria. Overall DGE rate after tumor enucleation was 5 % (95 % CI 2–10 %) based on six studies with a total amount of 231 included patients (see Fig. 5) [26, 28, 34, 35, 38, 46]. Overall DGE rate after distal pancreatectomy was 5 % (95 % CI 1–19 %, *I*^2^ 12 %) [34, 35, 46], based on three studies with a total of 62 included patients (see Fig. 6), after pancreatoduodenectomy 18 % (95 % CI 10–31 %, *I*^2^ 0 %) [34, 35, 46] based on three studies with a total of 51 included patients (see Fig. 7) and after central pancreatectomy, 16 % (95 % CI 1–71 %, *I*^2^ 73 %) [28, 34] (see appendix Fig. 17).

#### Postoperative hemorrhage

**Fig. 8:**
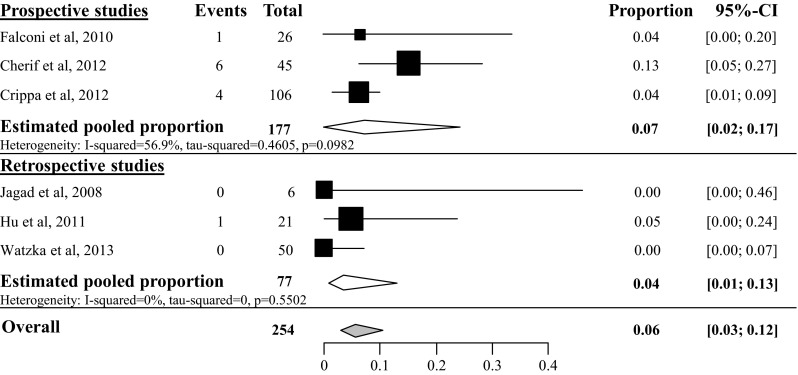
Overall postoperative hemorrhage rate after tumor enucleation

**Fig. 9:**
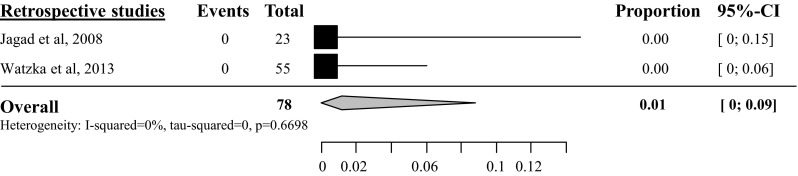
Overall postoperative hemorrhage rate after distal pancreatectomy

**Fig. 10:**
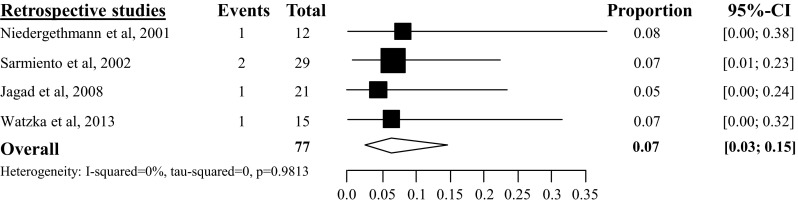
Overall postoperative hemorrhage rate after pancreatoduodenectomy

Postoperative hemorrhage was often not exactly defined according the ISGPS criteria in most studies. Therefore, a distinction between grade A and B/C hemorrhage could not be made. Six studies described the overall postoperative hemorrhage rate after tumor enucleation with a total amount of 254 included patients (see Fig. 8). In these studies, the overall postoperative hemorrhage rate was 6 % (95 % CI 3–12 %) [25, 26, 28, 35, 39, 44]. Two studies with a total amount of 62 included patients described an overall postoperative hemorrhage rate of 1 % after distal pancreatectomy (95 % CI 0–9 %, *I*^2^ 0 %) [35, 44] as depicted in Fig. 9. Overall postoperative hemorrhage rate after pancreatoduodenectomy was 7 % (95 % CI 3–15 %, *I*^2^ 0 %), based on four studies with a total of 77 included patients [35, 44, 47, 48] (see Fig. 10) and after central pancreatectomy 4 % (95 % CI 1–16 %, *I*^2^ 0 %), based on 2 studies (see appendix Fig. 18) [25, 28].

#### In-hospital mortality

Overall pooled in-hospital mortality rate after tumor enucleation was 3 % (95 % CI 2–5 %), based on 20 studies with a total amount of 624 patients [23–25, 28–40, 42, 44, 46] (see appendix Fig. 19). The overall pooled in-hospital mortality after distal pancreatectomy was 4 % (95 % CI 2–7 %) [23, 24, 29–37, 39, 42, 44, 45, 46], based on 16 studies with a total of 267 included patients (see appendix Fig. 20) and 6 % after pancreatoduodenectomy (95 % CI 3–12 %), based on 10 studies with a total of 146 included patients [23, 29–31, 34, 35, 44, 46–48] (see appendix Fig. 21). The overall pooled in-hospital mortality after central pancreatectomy was 4 % (95 % CI 1–16 %), based on 3 studies with a total of 51 included patients (see appendix Fig. 22) [25, 28, 34].

### Survival analysis

#### The 5-year overall and disease-specific survival in patients without liver metastases

**Fig. 11:**
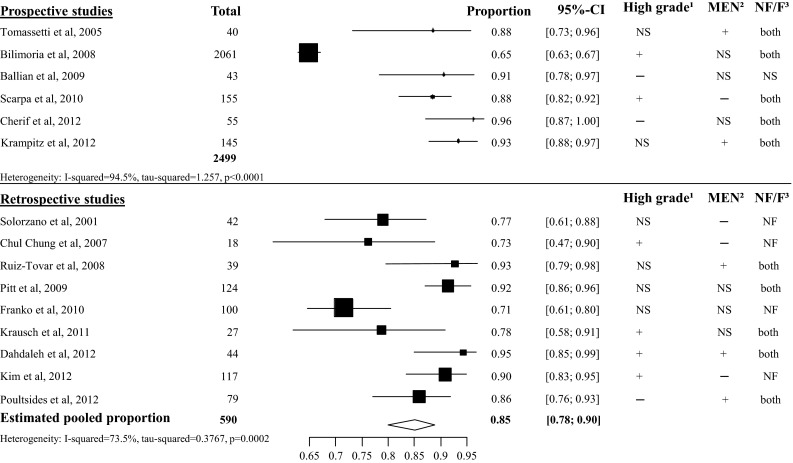
Overall 5-year survival in patients without liver metastases. ^1^ High grade: patients with grade 3 or poorly differentiated pNET may be included. ^2^ MEN: patients with a hereditary syndrome such as MEN1 syndrome or von Hippel Lindau may be included. ^3^ NF/F. Patients with non-functional pNET or functional pNET may be included. + Some patients are affected with the condition. − None of the patients are affected with the condition. NS not specified. The study did not specified the number of patients with the condition

In the survival analysis, a distinction is made between studies including patients with or without resected liver metastases. In the overall 5-year survival analysis of the resected patients without liver metastases, 15 studies were analyzed with a total of 3089 included patients [28, 36, 38, 41, 49–59]. The heterogeneity between the prospective studies was too high to perform a pooled meta-analysis (*I*^2^ 95 %), mainly caused by the study of Bilimoria et al. [59]. The estimated pooled proportion of the overall 5-year survival of the retrospective studies was 85 % (95 % CI 78–90 %, *I*^2^ 73.5 %), see Fig. 11. In the 5-year disease-specific survival (DSS) analysis, 6 studies were included with a total amount of 420 patients [43, 50, 52, 60–62]. The overall pooled 5-year DSS after pancreatic resection was 93 % (95 % CI 88–96 %), see appendix Fig. 23.

#### The 5-year overall and disease-specific survival in patients with liver metastases

**Fig. 12:**
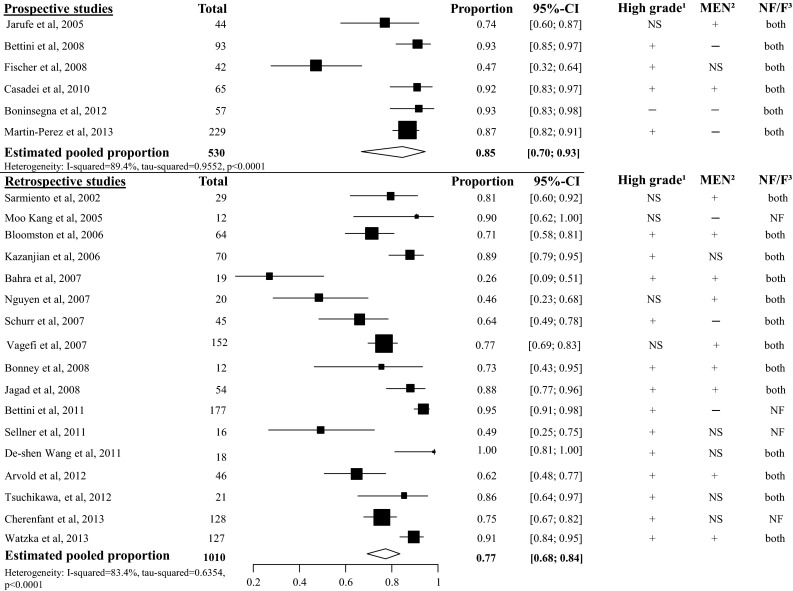
Overall 5-year survival in patients with liver metastases. ^1^ High grade: patients with grade 3 or poorly differentiated pNET may be included. ^2^ MEN: patients with a hereditary syndrome such as MEN1 syndrome or von Hippel Lindau may be included. ^3^ NF/F. Patients with non-functional pNET or functional pNET may be included. + Some patients are affected with the condition. − None of the patients are affected with the condition. NS not specified. The study did not specified the number of patients with the condition

In all the included studies, at least one patient per study had resected liver metastases. In the 5-year overall survival analysis, 23 studies were included with a total amount of 1540 patients [23, 35, 44, 46, 48, 63–80]. The heterogeneity was too high to perform an overall pooled proportion analysis, most studies included a proportion of high grade pNET (see Fig. 12). Four retrospective studies with a total of 207 included patients described the 5-year disease-specific survival in patients with liver involvement. The overall pooled 5-year DSS was 80 % (95 CI 66–90 %, *I*^2^ 70 %), see appendix Fig. 24 [81–84].

### Assessment of risk of bias

On overview of the risk of bias of all the included studies is listed in Table 1. The variety of the total points ranged from 5 to 12 points. None of the studies scored on unbiased assessment of the study endpoint or prospective calculation of the study size. Overall, 33/62 studies (53 %) had a high MINOR score of ≥10 and only 8 studies (13 %) had a low MINOR score ≤7.

**Table 1:** Risk of Bias according MINOR

Study	Inclusion C/S/B^a^	Clear stated aim	Inclusion consecutive patients	Prospective data collection	Endpoints appropriate to aim	Unbiased assessment of endpoint	Appropriate follow-up period	Loss to follow-up <5 %	Prospective calculation of study size	Total points
Niedergethmann et al. [47]	C	1	2	0	1	0	2	2	0	8
Solorzano et al. [53]	S	1	2	0	1	0	1	2	0	7
Chu et al. [81]	S	2	2	0	2	0	1	2	0	9
Matthews et al. [29]	C	2	2	0	2	0	2	2	0	10
Sarmiento et al. [48]	B	1	2	0	1	0	2	1	0	7
Guo et al. [31]	C	1	2	0	1	0	1	1	0	6
Norton et al. [30]	C	2	2	0	2	0	1	2	0	9
Jarufe et al. [23]	B	2	2	1	2	0	1	2	0	10
Kang et al. [68]	S	2	2	0	2	0	1	2	0	9
Sledzianowski et al. [45]	C	2	2	2	2	0	2	2	0	12
Tomassetti et al. [49]	S	2	2	2	2	0	2	2	0	12
Vagefi et al. [69]	S	2	2	0	2	0	2	1	0	9
Bloomston et al. [70]	S	2	2	0	2	0	2	2	0	10
Chung et al. [32]	C	1	2	0	1	0	2	2	0	8
Kazanjian et al. [46]	B	2	2	0	2	0	2	2	0	10
Winter et al. [82]	S	1	2	0	1	0	2	2	0	8
Bahra et al. [71]	S	2	2	1	2	0	2	2	0	11
Casanova et al. [33]	C	0	2	0	0	0	2	2	0	6
Chul Chung et al. [54]	S	2	2	0	2	0	1	2	0	9
Liu et al. [34]	C	2	2	0	2	0	2	2	0	10
Nguyen et al. [72]	S	2	2	0	2	0	2	2	0	10
Schurr et al. [73]	S	2	2	0	2	0	2	2	0	10
Bettini et al. [63]	S	2	2	2	2	0	2	2	0	12
Bilimoria et al. [59]	S	2	2	2	2	0	2	2	0	12
Bonney et al. [74]	S	1	2	0	1	0	1	1	0	6
Fernández-Cruz et al. [24]	C	2	2	2	2	0	2	2	0	12
Fischer et al. [64]	S	2	2	2	2	0	1	2	0	11
Jagad et al. [35]	B	1	2	0	1	0	2	2	0	8
Ruiz-Tovar et al. [36]	B	0	2	0	0	0	2	1	0	5
Ballian et al. [50]	S	2	2	2	2	0	2	1	0	11
Goh et al. [37]	C	2	2	0	2	0	2	1	0	9
Pitt et al. [38]	B	2	2	0	2	0	1	2	0	9
Casadei et al. [65]	S	2	2	1	2	0	2	2	0	11
Falconi et al. [25]	C	2	2	2	2	0	2	2	0	12
Franko et al. [55]	S	2	2	0	2	0	1	2	0	9
Goh et al. [60]	S	2	2	0	2	0	1	2	0	9
Ito et al. [83]	S	2	2	0	2	0	2	2	0	10
Pomianowska et al. [84]	S	2	2	0	2	0	2	2	0	10
Scarpa et al. [51]	S	2	2	2	2	0	2	2	0	12
Arvold et al. [78]	S	2	2	0	2	0	1	2	0	9
Bettini et al. [75]	S	2	2	0	2	0	2	2	0	10
Fernández-Cruz et al. [27]	C	2	2	2	2	0	2	2	0	12
Hu et al. [39]	C	2	2	0	2	0	2	2	0	10
Krausch et al. [56]	S	2	1	0	2	0	1	1	0	7
Sellner et al. [76]	S	2	2	0	2	0	2	2	0	10
Wang et al. [77]	S	2	2	0	2	0	2	1	0	9
Wang et al. [61]	S	2	2	0	2	0	2	2	0	10
Boninsegna et al. [66]	S	2	2	2	2	0	2	2	0	12
Cherif et al. [28]	B	2	2	2	2	0	2	2	0	12
Crippa et al. [26]	C	2	2	1	2	0	2	2	0	11
Dahdaleh et al. [57]	S	2	2	0	2	0	2	1	0	9
Inchauste et al. [40]	C	2	2	0	2	0	2	2	0	10
Kim et al. [58]	S	2	2	0	2	0	1	1	0	8
Krampitz et al. [52]	S	2	2	2	2	0	2	2	0	12
Poultsides et al. [41]	B	2	2	0	2	0	1	1	0	8
Tsuchikawa et al. [79]	S	2	2	0	2	0	1	1	0	8
Tsutsumi et al. [62]	S	2	2	0	2	0	2	2	0	10
Zhang et al. [42]	C	2	2	0	2	0	2	1	0	9
Cherenfant et al. [80]	S	1	2	0	2	0	1	1	0	7
Haugvik et al. [43]	B	2	2	0	2	0	2	2	0	10
Martin-Perez et al. [67]	S	2	2	2	2	0	1	1	0	10
Watzka et al. [44]	B	2	2	0	2	0	0	1	1	8

^a^Study included in *c* complication analysis, *s* survival analysis or *b* both complication and survival analysis

## Discussion

This is the first systematic review including a proportion analysis on postoperative complications, in-hospital mortality and 5-year survival in patients with a pancreatic neuroendocrine tumor. Pooled PF rate after tumor enucleation of the prospective studies was high (45 %) compared to overall pooled PF rate after distal pancreatectomy (14 %) and pancreatoduodenectomy (14 %). In patients with other diagnosis including pancreatic adenocarcinoma, the overall incidence of PF after pancreatoduodenectomy ranges from 2 % up to more than 20 % [85–88] and after distal pancreatectomy from 12–32 % [89–93] and the overall PF rate in non-pNET diagnosis is between 11 and 17 % compared to 6–34 % in patients with pNET [40, 45, 94–96]. This is coherent with the incidence of PF in patients with pNET in our review. Since the presence of PF accounts in the majority of cases for a prolonged hospital stay, the high incidence of these complications after tumor enucleation is alarming. Also the incidence of delayed gastric emptying (18 %) in patients with pNET after pancreatoduodenectomy in our review is comparable with the overall incidence from 14 to 45 % after pancreatoduodenectomy in patients with non-pNET [97–99].

The overall mortality in patients with pNET in our review is between 3 and 6 %. In the literature the recent overall mortality in non-pNET is between 0 and 4 % [91, 96, 100–102]. The overall in-hospital mortality rate in our review is slightly high. This is probably due to the fact that we included studies already from the year 2000 and centralization of pancreatic surgery takes place only since the last few years. The in-hospital mortality rate after pancreatoduodenectomy has been decreased from 15 % to even 1 or 2 % in high volume centers [103–105]. Therefore, the effect of centralization on in-hospital mortality is not shown in our review. Furthermore, in some studies on patients with pNET, pancreatic resection was not specified in pancreatoduodenectomy of distal pancreatectomy. In the analysis of the in-hospital mortality, these studies were excluded [10, 28, 40]. A second important point could be the texture of the pancreatic remnant after resection. In patients with pNET, especially small pNETs, there is no pancreatic duct dilation, no fibrosis and the pancreatic remnant is soft and viable. This is in contrast with most patients with non-pNET tumors with have a double duct sign and subsequent fibrosis of the pancreatic remnant. Since the texture of the pancreatic remnant is well known to be associated with PF and mortality this could a reasonable explanation [5, 106, 107]. Unfortunately, in this review, we could not find data on this important detail to draw conclusions concerning this point.

The analysis of postoperative complications in pancreatic surgery is more uniform since the clear definitions of these complications by the International Study Group of Pancreatic Surgery (ISGPS) [15–17]. The number of studies suitable for inclusion in the proportion analysis for pancreatic fistula grade B/C was limited. Most studies on grade B/C fistula (or delayed gastric emptying and postoperative hemorrhage) included patients with different underlying diseases. Patients with pNET were part of the studied cohort. These studies were not included in this review. Tumor enucleation is mainly indicated for pNET and therefore the number of studies for proportion analysis on grade B/C pancreatic fistula was relatively high compared to the other procedures (appendix Fig. 13). In future studies, we encourage the use of the ISGPS criteria in the analysis of postoperative complications and to describe the patients with pNET separately.

**Fig. 13:**
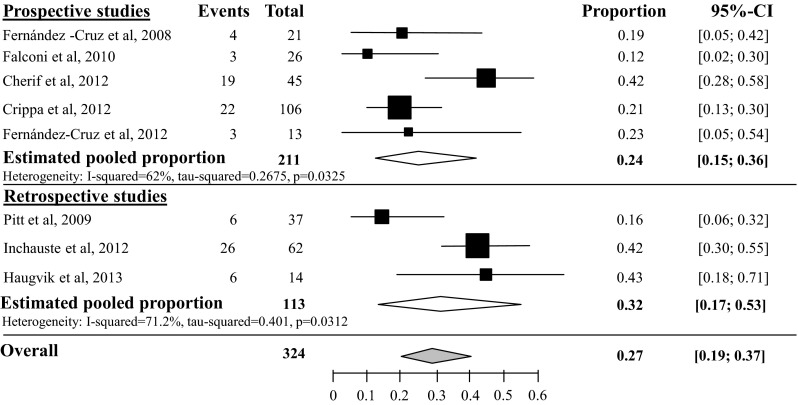
Pancreatic fistula rate grade B/C after tumor enucleation

Recently, Hüttner et al. described a high incidence of pancreatic fistula after tumor enucleation in patients with all types of pancreatic neoplasm [108]. Although the authors conclude that a tumor enucleation can be performed safely and is considerable instead of a standard resection, this conclusion should be interpreted with caution. Even in high volume centers, the incidence of pancreatic fistula was comparable after both tumor enucleation and standard resection (both 23 %). Although overall length of stay and mortality after tumor enucleation is lower compared to standard resection, patients with severe pancreatic fistula will have comparable length of stay and mortality. Since specialized care for patients with PF is important for overall outcome, enucleations should also be carried out in specialized centers.

A considerable amount of studies described the 5-year survival after pancreatic resection with or without liver metastases. The 5-year disease-specific survival in patients with and without liver metastases was fairly comparable with, respectively, 93 and 80 %. Although there will be differences in tumor differentiation, functionality, or hereditary tumors, the survival rate after surgical resection in patients with liver metastases is high. An aggressive treatment in patients with liver metastases may be justified. However, both patients and tumor characteristics, such as total tumor load in the liver, are important in this treatment. In our review, the heterogeneity between the included studies in the 5-year overall survival analysis was high (Figs. 9 and 10). These differences can be explained by the patients’ characteristics of the included studies. For example, in the study of Bahra et al., patients were enrolled with at least two malignant factors such as invasion in adjacent organs, metastases, tumor invasion, tumor size ≥2 cm, and tumor grade 2 or 3 pNET [71]. Bilimoria et al. [59] also enrolled patients with distant metastases (20 %), positive lymph nodes (52.8 %), and poorly differentiated pNET (22.1 %). Most likely, a high grade/poorly differentiated tumor has more influence on survival than the presence of resected liver metastases. This hypothesis has not been analyzed in this review. In addition, in most studies no differentiation was made between functional and non-functional pNET.

Since no randomized controlled trials were available, heterogeneity was notable. During full-text analysis, some studies were not clear or incomplete on the description of the outcome. For example, studies described the postoperative after “standard pancreatic resection” but different definitions for a standard resection were used. Some studies described patients after pancreatoduodenectomy and distal pancreatectomy [10, 28, 108] while other studies described patients with all types of pancreatic resection including central pancreatectomy and total pancreatectomy [26, 38, 40]. Furthermore, some large studies, especially studies that extracted the data from the SEER database, described the survival outcome per tumor stage and most of these studies have not described an overall 5-year survival. Moreover, it was not always clear if all the included patients with stage IV disease were operated. All these studies were excluded from this review. There is no agreement of the exact cut-off value of heterogeneity in which it is accepted to perform a meta-analysis. According to the Cochrane handbook, with an *I*^2^ above 75, heterogeneity is considerable [21]. By the strict inclusion criteria, effort has been made to include homogeneous data and studies with good quality but the diversity of the studies on pNET is considerable and this review shows the best available data up till now.

## Conclusion

Based on this review, we would like to recommend using uniform definitions for “pancreatic resection” or well-described “atypical resections” for a careful comparison of clinical outcome. Furthermore, the ISGPS criteria and Clavien–Dindo grading system should be used in the analysis of postoperative complications. In survival analysis, distinguishes should be made between tumor grade/tumor differentiation, patients with a hereditarily syndrome and patients with a functional or non-functional pNET. Although pNET is a rare disease, studies on postoperative outcome and survival must be uniform and clear to be able to interpret the results in the right way and to use the results in daily practice.

## Figures and Tables

**Fig. 14:**
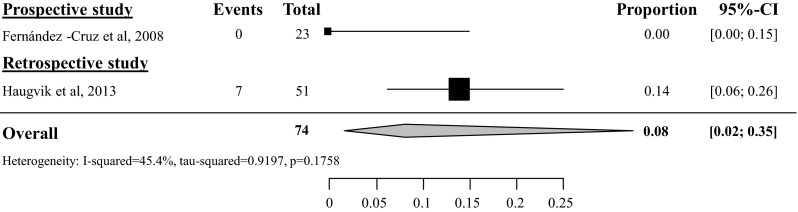
Pancreatic fistula rate grade B/C after distal pancreatectomy

**Fig. 15:**
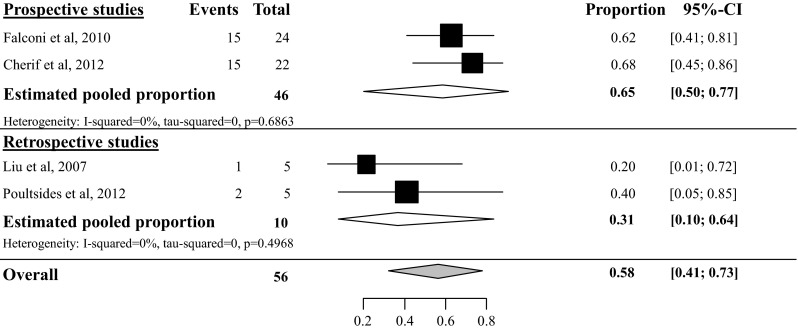
Overall pancreatic fistula rate after central pancreatectomy

**Fig. 16:**
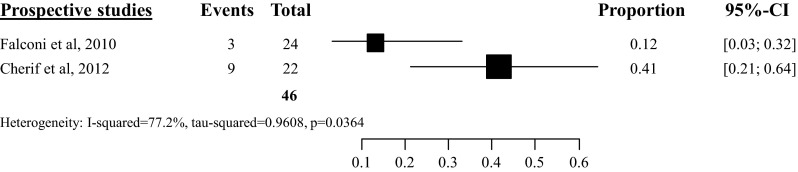
Pancreatic fistula rate grade B/C after central pancreatectomy

**Fig. 17:**
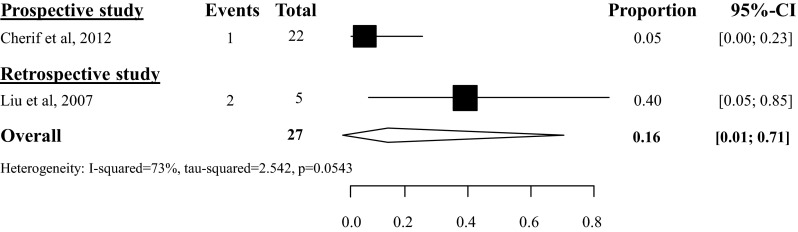
Overall delayed gastric emptying rate after central pancreatectomy

**Fig. 18:**
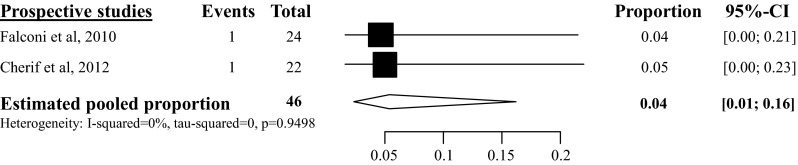
Overall postoperative hemorrhage rate after central pancreatectomy

**Fig. 19:**
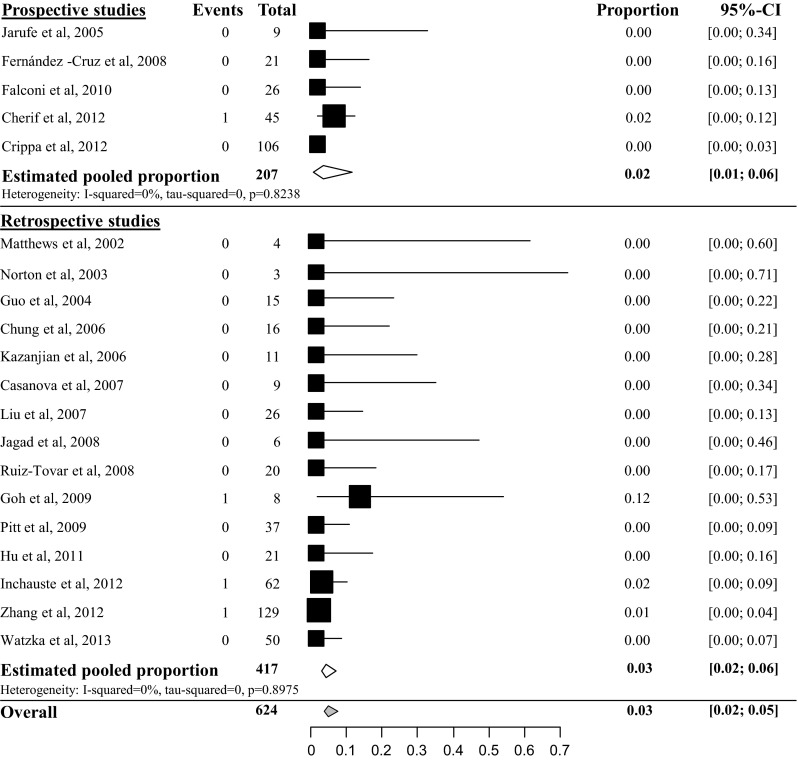
In-hospital mortality rate after tumor enucleation

**Fig. 20:**
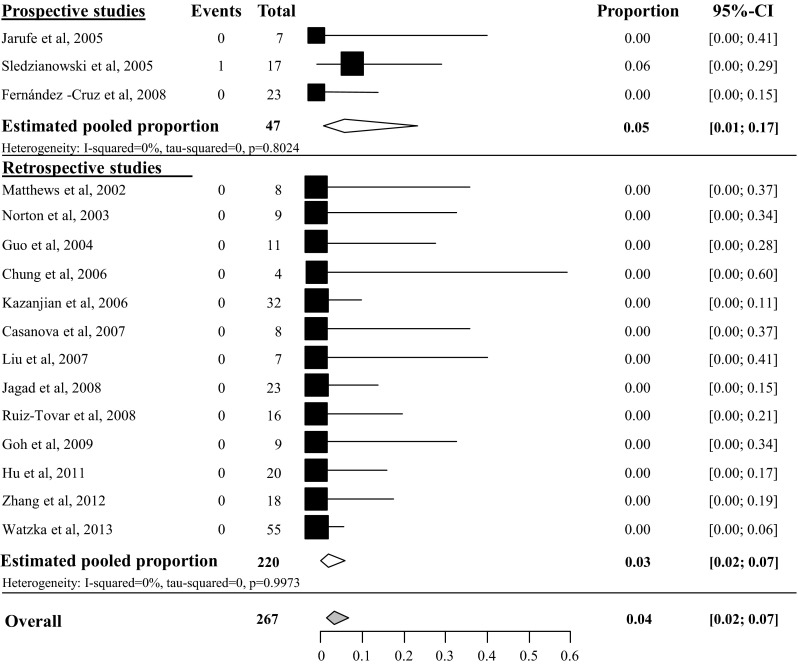
In-hospital mortality rate after distal pancreatectomy

**Fig. 21:**
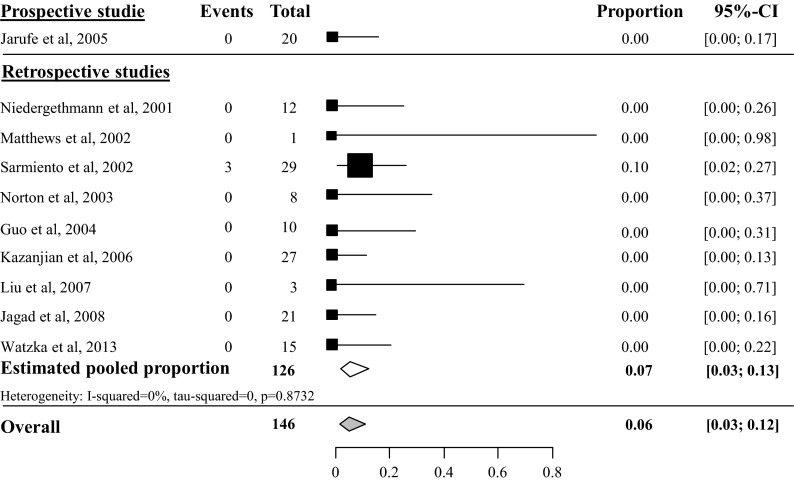
In-hospital mortality rate after pancreatoduodenectomy

**Fig. 22:**
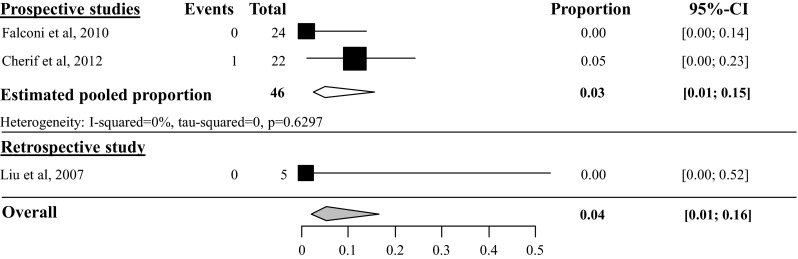
In-hospital mortality rate after central pancreatectomy

**Fig. 23:**
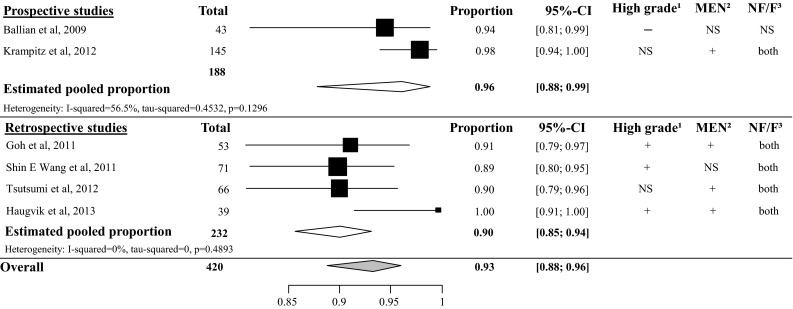
5-year disease-specific survival in patients without liver metastases. ^1^ High grade: patients with grade 3 or poorly differentiated pNET may be included. ^2^ MEN: patients with a hereditary syndrome such as MEN1 syndrome or von Hippel Lindau may be included. ^3^ NF/F. Patients with non-functional pNET or functional pNET may be included. + Some patients are affected with the condition. − None of the patients are affected with the condition. NS not specified. The study did not specified the number of patients with the condition

**Fig. 24:**
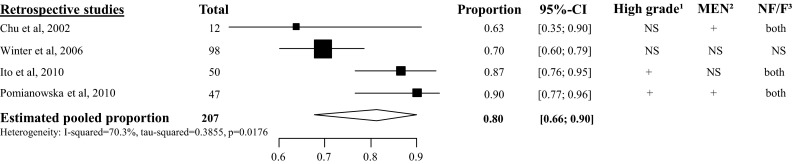
5-year disease-specific survival in patients with liver metastases. ^1^ High grade: patients with grade 3 or poorly differentiated pNET may be included. ^2^ MEN: patients with a hereditary syndrome such as MEN1 syndrome or von Hippel Lindau may be included. ^3^ NF/F. Patients with non-functional pNET or functional pNET may be included. + Some patients are affected with the condition. − None of the patients are affected with the condition. NS not specified. The study did not specified the number of patients with the condition

## Appendix

See Figs. 13, 14, 15, 16, 17, 18, 19, 20, 21, 22, 23, 24.
